# Thoron Interference on Performance of Continuous Radon Monitors: An Experimental Study on Four Devices and a Proposal of an Indirect Method to Estimate Thoron Concentration

**DOI:** 10.3390/ijerph19042423

**Published:** 2022-02-19

**Authors:** Christian Di Carlo, Marco Ampollini, Sara Antignani, Mario Caprio, Carmela Carpentieri, Francesco Bochicchio

**Affiliations:** Italian National Institute of Health, National Center for Radiation Protection and Computational Physics, Viale Regina Elena, 299-00161 Rome, Italy; marco.ampollini@iss.it (M.A.); sara.antignani@iss.it (S.A.); mario.caprio@iss.it (M.C.); carmen.carpentieri@iss.it (C.C.)

**Keywords:** radon monitor, thoron interference, indoor radon concentration, thoron sensitivity

## Abstract

The performance of continuous radon monitors (CRMs) is usually evaluated under controlled conditions in a radon chamber during calibrations or intercomparison exercises. The impact of thoron on CRMs response is rarely evaluated; in case the evaluation is performed, it is carried out in a controlled atmosphere with relatively constant, homogeneous, and generally high thoron concentrations and very low radon levels. In a real indoor environment, both radon and thoron concentrations are extremely variable, so the thoron interference evaluations reported in the literature are generally not applicable to CRMs used to measure radon concentration indoors. For this reason, an experimental study was carried out with four different CRMs in an indoor environment (an office room) where medium-to-high concentrations of both radon and thoron were expected. Thoron concentration has been separately evaluated throughout two different active monitors. Three CRMs resulted in overestimations of radon concentration by about 10% due to thoron interference, whereas such interference results were negligible for the fourth CRM. However, the thoron interference can also be used to assess thoron concentration by using CRM not specifically designed to do so. Based on the results of this study, an indirect method to assess thoron concentration is indeed proposed, relying on the combination of two identical monitors (one placed right close to the wall and the other one far enough from there).

## 1. Introduction

Continuous radon monitors (CRMs) are being increasingly used in many application fields including the evaluation of short- and long-term effectiveness of remedial actions and the estimation of the actual exposure of workers [1]. Similar studies need active measurements in some to many premises, working places, or dwellings, and only the recent appearance on the market of inexpensive CRMs has made this kind of survey affordable.

However, the performance of any detectors, including the affordable CRMs, needs to be evaluated in terms of accuracy, precision, and response linearity. Generally, such evaluations are carried out throughout calibrations or intercomparison exercises performed in a radon chamber [2,3,4]. Some intercomparisons highlighted the so-called thoron (in the following, also referred to as Tn) interference on CRMs. The latter realizes when the returned radon (in the following also referred to as Rn) concentration is overestimated due to the non-negligible thoron concentration. This phenomenon, as expected, has been observed especially close to the walls, where the thoron concentration is generally maximum.

The evaluation of thoron interference is generally performed by measuring the radon concentration by CRMs in a controlled atmosphere with fixed and generally high thoron concentrations and very low radon levels [2]. Such interference has been observed to be significant, especially for those CRMs operating in flux mode [5]—i.e., radon enters the chamber in a pumped air flux—and with no spectrometric analysis capabilities for alpha particles emitted by radon and thoron progenies. However, the response of CRMs operating in diffusion mode—i.e., radon enters the chamber by molecular diffusion—has been observed to be affected too when the thoron concentration is high. High thoron concentrations are far from rare in south-central Italy (for example, due to the vast usage of tuff and pozzolana as building materials) [6]. Similarly, detectors capable of performing alpha spectrometry have been reported to evidence thoron interference due to energy resolution and algorithm used to calculate the radon concentration [4]. Specific correction algorithms have been conceived to avoid overestimation of radon concentration due to thoron in measurements performed by CRMs operating energy discrimination [1] or not [7].

The primary purpose of this work is to evaluate the interference introduced by thoron presence on the radon concentration returned by two reference high-cost CRMs and two cheap CRMs exposed in the same atmosphere where radon and thoron coexist with levels of the same order of magnitude.

## 2. Materials and Methods

The experiments designed to evaluate the thoron interference have been performed on four different CRMs types manufactured by three different producers (Table 1). Three detectors are pulsed ion chambers, but the filling gas is air, so they cannot perform spectrometry of alpha particles emitted by radon and thoron progenies. One of the chosen detectors has a semiconductor (i.e., silicon) as a sensitive element placed on the bottom of a chamber where an electrical field drives charged radon and thoron progenies to the sensor. Thus, it can perform alpha spectrometry of Rn and Th daughters.

Only the AlphaGUARD detectors (both the PQ2000 Pro and D50 models) have been calibrated by the manufacturer before being delivered.

Two replicates of each CRMs type have been used in the experiments carried out.

The experiment has been articulated in three distinct and subsequent phases.

### 2.1. Intercomparison between Detectors Placed at the Room Center, with Negligible Thoron Concentration

During this first phase, the response of the CRMs has been tested in a near-zero thoron concentration atmosphere, as it is reasonable to be assumed far from room walls [8]. So, for seven days, the four couples of detectors (i.e., two replicates for each CRM type) have been placed more than 150 cm far from the nearest wall for seven days.

This initial exposure has also been necessary to evaluate the accuracy of TESLA and FTLAB detectors, whose calibration certificate was not provided by the manufacturer.

During this experiment, the AlphaGUARD detectors have been assumed as reference monitors. The reference radon concentration has been assumed to be the arithmetic mean of the weekly-averaged radon concentrations returned by the four devices.

### 2.2. Evaluation of Thoron Interference for the Four Detectors Types

**Figure 1:**
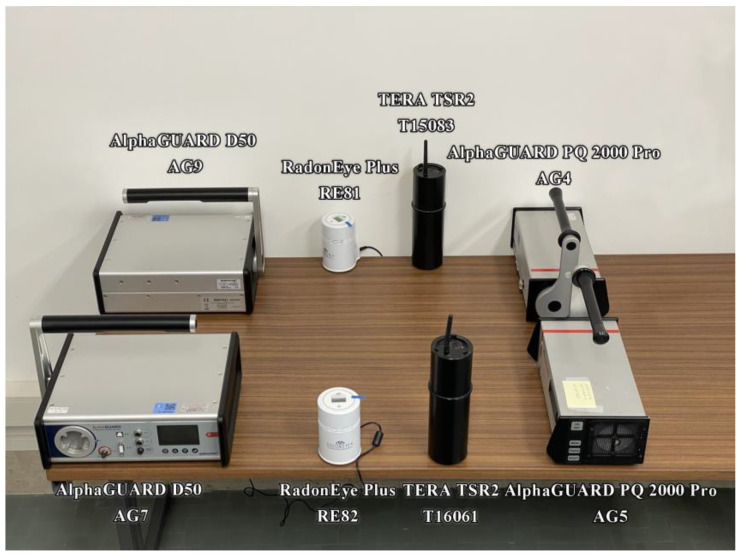
Positions, relative to the wall, of the four couples of CRMs during the first seven-days lasting exposure.

One replicate for each CRM type has been placed in close contact, about 2 cm, to a wall previously demonstrated to exhale thoron highly. The second replicate has been positioned 80 cm far from the same wall (Figure 1).

TERA TSR2 and RadonEye Plus work only in diffusion mode, so the AlphaGUARDs, which in principle can operate both in diffusion and flow mode, have been operated analogously to avoid misleading results due to different sampling techniques.

Two subsequent exposures have been realized, each lasting seven days to avoid misleading results due to periodic short-term fluctuations of radon and thoron concentrations (mainly due to temperature and pressure trends and occupancy patterns of the room). During the second one, at similar Rn and Tn concentrations, the position of the two detectors of each kind has been switched. Longer exposures have been excluded in order to avoid climatic changes to have effects on radon and thoron concentration indoor.

For each monitor, data have been downloaded and the thoron interference has been estimated relying on thoron measurements performed at 2 cm from the wall by two distinct detectors: RAD7 (manufactured by Durridge) and AlphaGUARD DF2000 (manufactured by Saphymo). Considering that both the devices work in flow mode, thoron measurements have been performed right after the exposures and not during them to avoid introducing interferences in the atmosphere sampled by the eight CRMs tested.

## 3. Results

The intercomparison results between detectors placed at the center of the room are shown in Table 2. The significance of the difference between measurements carried out by the two replicates of each detector has been evaluated through the Student’s t-test assuming a significance level of 5%. The agreement between the replicates of the same detector type is good (except for TERA-TSR2, whose measurements show a statistically significant difference).

The average radon concentration obtained with RadonEye plus and TERA TSR2 significantly differs from AlphaGUARD (both D-50 and PQ 2000 Pro model). Thus, new calibration factors have been computed for RadonEye Plus and TERA TSR2, assuming the AlphaGUARD detectors as reference units.

The results of the second phase (i.e., the evaluation of thoron interference for the four detector types) are shown in Table 3 and Table 4, the latter reporting results of the second experiment in which the positions of the replicates of each detectors couple have been switched. During the first experiment, AlphaGUARD D50 and PQ 2000 Pro results have been very close as it is reasonable considering that the two models use the same radon chamber. For such a reason, the AlphaGUARD PQ 2000 Pro has been excluded from the second experiment.

**Figure 2:**
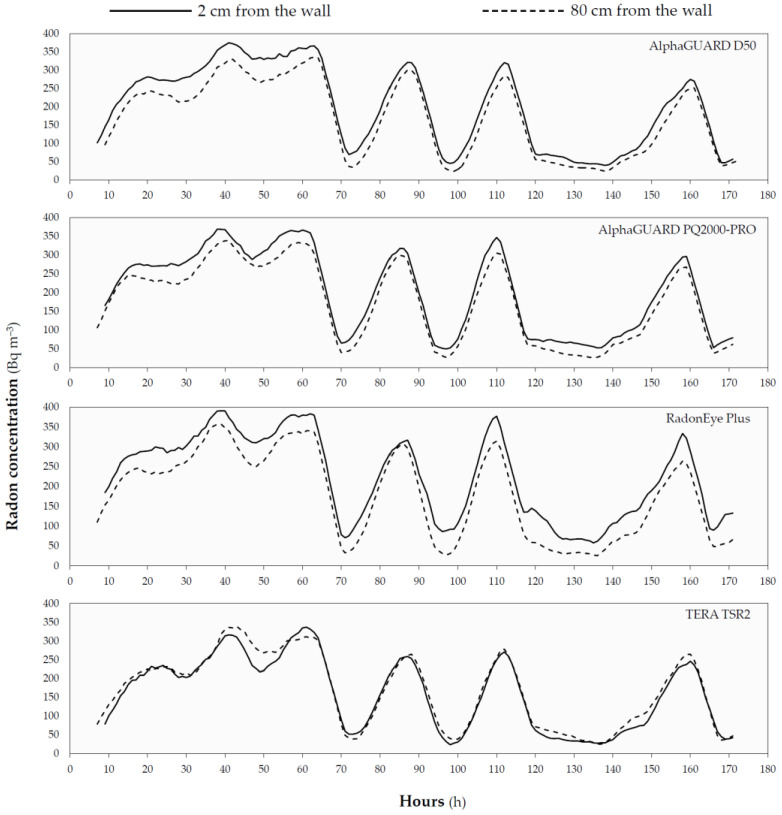
Radon concentration measured during the first experiment at 2 cm (full line) and 80 cm (dashed line) for the four replicates of CRMs considered.

Figure 2 reports the radon concentrations trends measured during the first seven-day-lasting measurement performed by the eight CRMs considered (four at 2 cm and the remaining at a distance of 80 cm from the wall).

## 4. Discussion

The calibrated AlphaGUARDs D50 (i.e., AG7 and AG9, respectively) positioned at the center of the room during the first and the second experiment of the second phase, have returned almost identical seven-days-average radon concentration, demonstrating that the two experiments of phase two (Table 3 and Table 4) have been carried out with about the same radon concentration in the room.

The difference between the radon concentration measured 80 cm and 2 cm far from the wall is statistically significant for AlphaGUARD and RadonEye Plus. The second experiment returned almost identical results in terms of absolute and relative difference, (Table 4), suggesting that: (a) the differences cannot be attributed to anything different than the thoron interference; and (b) thoron concentration at 2 cm from the wall is about the same during the two experiments.

Concerning TERA TSR2, the difference between radon measured in the two positions is not statistically significant in both experiments (Table 3 and Table 4); the thoron presence seems not to affect radon concentration measured.

Figure 2, illustrating radon concentration trends during the first experiment of the second phase, shows that the difference between seven-days-averaged radon concentration seen for AlphaGUARD and RadonEye Plus is due to a systematic bias from the concentration measured close to and far from the wall.

Measurements of thoron at 2 cm from the wall have finally been performed to evaluate the actual thoron concentration in the position where the thoron interference is evaluated. Such measurements were performed at the end of the second phase experiments and lasted one day each. The results are shown in Table 5.

The thoron concentration measured by RAD7 is systematically less (i.e., about 40% than the corresponding value measured by the AlphaGUARD DF2000). However, for both devices, the value measured right after the first experiment does not differ statistically significantly from the one obtained after the second experiment. This agrees with the lower indoor variability of thoron concentration with respect to the radon one and the measuring point chosen and fixed [8]. Given all this, the thoron concentration has been reasonably assumed constant and equal during the first and the second tests.

**Figure 3:**
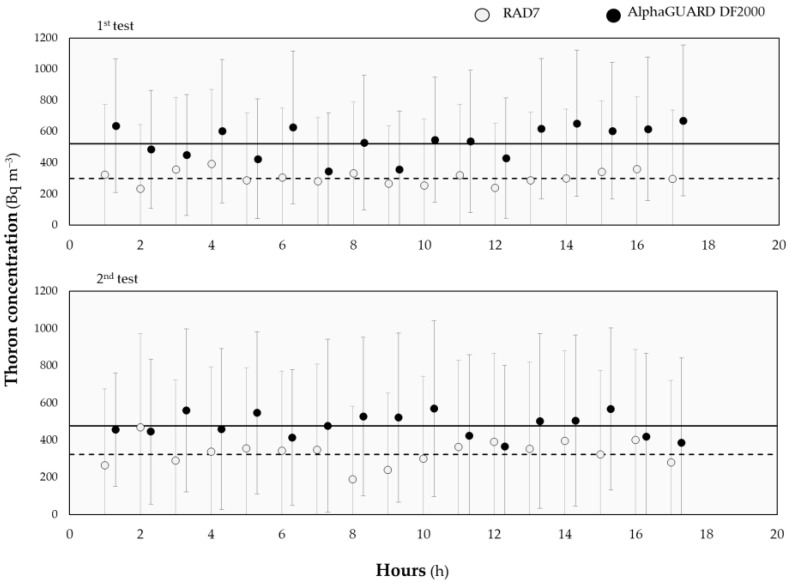
Comparison between thoron concentration measured by RAD7 (empty bullets) and AlphaGUARD DF2000 (full bullets) at 2 cm from the wall after the first (upper graph) and the second (lower graph) interference test. The plain and dashed lines stand for the arithmetic mean of returned values by the AlphaGUARD DF2000 and RAD7, respectively. All results are reported with uncertainty (with coverage factor = 2).

Considering the bias existing between measurements performed by the RAD7 and the AlphaGUARD DF2000 and the lack of *a-priori* reasons to predilect one or the other device, the arithmetic mean of the values returned in the two tests by the two instruments has been assumed as the reference thoron concentration to be used in a first quantitative estimation of the thoron interference, i.e., 406 ± 51 Bq m^−3^. The full trends measured by the two instruments are reported in Figure 3.

Values of thoron interference, $I_{\mathrm{Tn}}$, have been computed as:

(1) $$I_{\mathrm{Tn}}=\frac{C_{\mathrm{Rn},2\;\mathrm{cm}}-C_{\mathrm{Rn},80\;\mathrm{cm}}}{C_{\mathrm{Tn},mean}}$$

where:

$C_{\mathrm{Rn},2\;\mathrm{cm}}$ is the seven-days mean of the radon concentration measured 2 cm from the wall;

$C_{\mathrm{Rn},80\;\mathrm{cm}}$ is the seven-days mean of the radon concentration measured 80 cm from the wall;

$C_{\mathrm{Tn},\mathrm{mean}}$ is the mean thoron concentration, obtained by averaging the results obtained during the two tests by both measuring instruments (i.e., RAD7 and AlphaGUARD DF2000).

The interference has been evaluated only for AlphaGUARD DF50 and RadonEye Plus detectors by considering the radon concentration difference observed during the first experiment. The values are reported in Table 6. AlphaGUARD PQ2000 Pro and TERA TSR 2 detectors have been excluded since, for the first, no statistically significant difference has been observed with the D50 model and, for the second, no interference has been observed at all.

For the AlphaGUARD monitor, the estimated values of thoron interference are in very good agreement with similar literature findings (i.e., 10%) [5,9,10,11]. Pertaining to RadonEye Plus, to the authors knowledge, the results of similar evaluations have not been previously published.

During the experiments, the room hosting the measurements and the adjacent ones have been used according to the usual occupancy pattern. As a consequence, the ventilation of inner and outer room air significantly changed over the days mainly due to window and door openings. Previous works have shown that thoron interference can vary significantly for diffusive CRMs due to ventilation changes [11]. Further measurements are desirable in order to study such changes and provide ventilation-dependent thoron interference values.

### Proposal of an Indirect Method to Estimate Thoron Concentration

Active monitors used to measure thoron concentration indoor are generally expansive mainly since active sampling is generally required (i.e., throughout a pump), and counts due to thoron and its progeny need to be distinguished from those due to radon and its progeny.

However, on the basis of the results of this study, the thoron interference ($I_{\mathrm{Tn}}$) affecting continuous radon monitors can be exploited to give a first estimation of thoron concentration also by using of devices not specifically designed to measure thoron concentration. This can be carried out by two identical devices (i.e., same operating principle but also the same model) and by placing one at the position where thoron concentration is intended to be estimated and the other one far enough from likely thoron sources (i.e., walls or soil). The thoron estimation can be obtained as follows:

$$C_{\mathrm{Tn},\;x}C_{\mathrm{Tn},\;x}=\frac{C_{\mathrm{Rn},\;x}-C_{\mathrm{Rn},\;\mathrm{Tn}\approx 0}}{I_{\mathrm{Tn}}}$$

where:

$C_{\mathrm{Rn},\;x}$ is the radon concentration measured at the position *x*, where thoron concentration wants to be estimated;

$C_{\mathrm{Rn},\;\mathrm{Tn}\approx 0}$ is the radon concentration measured at the same room as $C_{\mathrm{Rn},\;x}$ but far enough from likely thoron sources. Here, the thoron concentration has to be assumed equal to zero;

$C_{\mathrm{Tn},x}C_{\mathrm{Tn},mean}$ is the thoron concentration estimated at the position *x*.

$C_{\mathrm{Rn},x}$ and $C_{\mathrm{Rn},\;\mathrm{Tn}\approx 0}$ can be either single measurements (i.e., returned by the continuous radon monitors after one measuring interval), or averaged values over more than one measuring intervals. If single measurements are being used, attention should be paid to avoid the transient characterizing the chamber filling right after having moved the detector from a room to another. This issue is particularly critical for CRMs operating in diffusion mode.

The accuracy of a similar evaluation is strongly dependent on the calibration of both the detectors employed. However, the discussed method would be not sensitive to inaccuracy of detectors caused by the same bias (e.g., detectors with a similar background grown through the years). The precision of the method depends on (*i*) the sensitivity of detectors used, (*ii*) the integration time, and (*iii*) the thoron interference $I_{\mathrm{Tn}}$.

## 5. Conclusions

Interference due to thoron has been observed to determine an overestimated radon concentration returned by some continuous radon monitors. An interference of about 10% has been observed for AlphaGUARD monitors (regardless of the model) and the RadonEye Plus monitor, whereas the interference has resulted to be negligible on TERA TSR2 detectors.

The scientific literature contains several examples of indoor thoron concentration close to the walls in the range 500–1000 Bq m^−3^ or higher. In similar cases, using CRMs with thoron interference of 10% would result in an overestimation of 50–100 Bq m^−3^ or higher. Consequently, attention should be paid to position of monitor, especially in case of buildings made of building materials with considerable thoron exhalation (e.g., tuff and pozzolana). This issue is particularly critical for those CRMs that need electrical power supply from the net (i.e., no integrated battery), since these monitors are more likely to be positioned close to the walls, where electric sockets are installed.

The relatively high value of thoron interference experienced by the RadonEye Plus monitor, together with its low cost (i.e., some hundreds of euros) and the high sensitivity (i.e., 81 cph per 100 Bq m^−3^), opens the way to the possibility to use such monitors to give a first, rapid and inexpensive estimation of the thoron concentration at a certain position indoor. Two replicates of the same CRM are necessary for this purpose: one placed where thoron concentration wants to be estimated and the other one far enough from likely thoron source to assume its concentration very low (i.e., ≈ 0 Bq m^−3^).

## Figures and Tables

**Table 1:** List of continuous radon monitors considered in the experiments aiming to evaluate the thoron interference. To evaluate the overall uncertainty, other than the stochastic component the systematic uncertainty should be considered (it is generally about 5%).

Detector	Model	Manufacturer	Operating Principle	Detection Efficiency	Stochastic Uncertainty ^a^
				cph per 100 Bq m^−3^	
AlphaGUARD	PQ2000 Pro	Saphymo	Pulsed Ion Chamber	300	6%
AlphaGUARD	D50	Saphymo	Pulsed Ion Chamber	300	6%
Tera	TSR2	TESLA	Semiconductor	25	20%
RadonEye+	RD200P	FTLAB	Pulsed Ion Chamber	81	11%

^a^ Coverage factor k=1.

**Table 2:** Results of intercomparison realized far from walls in order to compare the response of the continuous radon monitors in near-zero thoron atmosphere.

Detector Type	Device ID	*C*_Rn_	Standard Error	95% CI
		Bq m^−3^	Bq m^−3^	Bq m^−3^
AlphaGUARD-D50	AG9	220	2	(215–225)
AlphaGUARD–PQ 2000 Pro	AG4	227	2	(223–230)
	AG5	227	3	(222–232)
RadonEye Plus	RE82	241	3	(235–247)
	RE81	247	3	(241–253)
TERA-TSR2	T15083	202	3	(195–208)
	T16061	218	3	(211–225)

**Table 3:** Results of the first seven-days measurement realized by placing, for each couple of detector type, one replicate very close to the wall (i.e., 2 cm) and the other one at a distance of 80 cm. *C*_Rn, 80 cm_ is the seven-days mean of the radon concentration measured 80 cm from the wall and the *C*_Rn cm_ is the same mean computed from results of the same replicate placed close to the wall. All results are reported with uncertainty (with coverage factor = 2).

Detector Type	*C*_Rn, 80-cm_	*C*_Rn, 2 cm_	*C*_Rn,2 cm_–*C*_Rn, 80-cm_	95% CI
	Bq m^−3^	Bq m^−3^	Bq m^−3^	Bq m^−3^
AlphaGUARD-D50	165 ± 9	198 ± 9	33 ± 13	(8–58)
AlphaGUARD–PQ 2000 Pro	170 ± 9	198 ± 9	28 ± 12	(3–53)
RadonEye Plus	175 ± 9	220 ± 9	45 ± 13	(20–70)
TERA-TSR2	157 ± 6	165 ± 8	9 ± 10	(−11–29)

**Table 4:** Results of the second seven-days measurement realized by switching the position of replicates of each detectors couple with respect to the position of the first experiment discussed in Table 3. C_Rn, 80 cm_ is the seven-days mean of the radon concentration measured 80 cm from the wall and the C_Rn, 2 cm_ is the same mean computed from results of the same replicate placed close to the wall. All results are reported with uncertainty (with coverage factor = 2).

Detector Type	C_Rn, 80-cm_	C_Rn__,2 cm_	C_Rn__,2 cm_–C_Rn, 80-cm_	95% CI
	Bq m^−3^	Bq m^−3^	Bq m^−3^	Bq m^−3^
AlphaGUARD-D50	162 ± 8	193 ± 8	31 ± 11	(9–54)
RadonEye Plus	176 ± 8	220 ± 8	44 ± 12	(21–68)
TERA-TSR2	168 ± 6	162 ± 7	−6 ± 11	(−28–15)

**Table 5:** Results of the second seven-days measurement realized by switching the position of replicates of each detectors couple with respect to the position of the first experiment discussed in Table 3. C_Rn, 80 cm_ is the seven-days mean of the radon concentration measured 80 cm from the wall and the C_Rn, 2 cm_ is the same mean computed from results of the same replicate placed close to the wall. All results are reported with uncertainty (with coverage factor = 2).

Detector Type	C_Tn, 1st_	C_Tn__, 2nd_	C_Tn__, mean_
	Bq m^−3^	Bq m^−3^	Bq m^−3^
AlphaGUARD DF200	521 ± 102	476 ± 103	499 ± 72
RAD7	302 ± 106	324 ± 108	313 ± 76
Mean (AlphaGUARD DF200 RAD7)			406 ± 51

**Table 6:** Estimations of thoron interference. *C*_Tn, mean_ is obtained by averaging the results obtained during the two tests by both measuring instruments. *C*_Rn, 80 cm_ is the seven-days mean of the radon concentration measured 80 cm from the wall and the *C*_Rn cm_ is the same mean computed from results of the same replicate placed close to the wall.

Detector Type	*C*_Tn_ _, mean_	*C*_Rn__, 2 cm_ – *C*_Rn, 80-cm_	Thoron Interference
	Bq m^−3^	Bq m^−3^	%
AlphaGUARD-D50	406 (313–499)	32 ± 8	8% (6%–10%)
RadonEye Plus	406 (313–499)	45 ± 9	11% (9%–14%)

## Data Availability

The data presented in this study are available on request from the corresponding author.
